# Customizable Ligand Exchange for Tailored Surface Property of Noble Metal Nanocrystals

**DOI:** 10.34133/2020/2131806

**Published:** 2020-01-21

**Authors:** Qikui Fan, Hao Yang, Juan Ge, Shumeng Zhang, Zhaojun Liu, Bo Lei, Tao Cheng, Youyong Li, Yadong Yin, Chuanbo Gao

**Affiliations:** ^1^Frontier Institute of Science and Technology, State Key Laboratory of Multiphase Flow in Power Engineering, Xi'an Jiaotong University, Xi'an, Shaanxi 710054, China; ^2^Institute of Functional Nano & Soft Materials (FUNSOM), Jiangsu Key Laboratory for Carbon-Based Functional Materials & Devices, Joint International Research Laboratory of Carbon-Based Functional Materials and Devices, Soochow University, Suzhou, Jiangsu 215123, China; ^3^Department of Chemistry, University of California, Riverside, California 92521, USA

## Abstract

It is highly desirable, while still challenging, to obtain noble metal nanocrystals with custom capping ligands, because their colloidal synthesis relies on specific capping ligands for the shape control while conventional ligand exchange processes suffer from “the strong replaces the weak” limitation, which greatly hinders their applications. Herein, we report a general and effective ligand exchange approach that can replace the native capping ligands of noble metal nanocrystals with virtually any type of ligands, producing flexibly tailored surface properties. The key is to use diethylamine with conveniently switchable binding affinity to the metal surface as an intermediate ligand. As a strong ligand, it in its original form can effectively remove the native ligands; while protonated, it loses its binding affinity and facilitates the adsorption of new ligands, especially weak ones, onto the metal surface. By this means, the irreversible order in the conventional ligand exchange processes could be overcome. The efficacy of the strategy is demonstrated by mutual exchange of the capping ligands among cetyltrimethylammonium, citrate, polyvinylpyrrolidone, and oleylamine. This novel strategy significantly expands our ability to manipulate the surface property of noble metal nanocrystals and extends their applicability to a wide range of fields, particularly biomedical applications.

## 1. Introduction

Colloidal noble metal nanocrystals have received extensive interest due to their broad applications in catalysis [1–4], electronics [5, 6], biosensing [7–9], imaging [10, 11], and medicine [12, 13]. In general, these colloidal nanocrystals are obtained as inorganic-organic hybrids from a wet chemical synthesis, each containing a metallic core and a layer of organic capping ligands on the surface [14, 15]. The capping ligands are indispensable to maintain the colloidal dispersity of the particles. In addition, they regulate the growth of the nanocrystals into specific morphology by selectively adsorbing on certain facets of the nanocrystals [16–18]. Therefore, conventional syntheses of shape-controlled noble metal nanocrystals are always assisted by deliberately selected capping ligands, which are, however, not necessarily desired for the target applications. For example, oleylamine (OAm) and polyvinylpyrrolidone (PVP) are widely used capping ligands due to their strong adhesion to the metal surface, but they block the catalytically active sites or electromagnetic hotspots of the nanocrystals and significantly reduce their activities in catalysis and surface-enhanced Raman scattering [19, 20]. Many capping ligands such as cetyltrimethylammonium bromide (CTAB) are biotoxic, which is an obvious obstacle for the use of these noble metal nanocrystals in biological applications [21, 22]. Additionally, in some investigations, the surface property of the nanocrystals should be kept consistent for an unambiguous assessment of their performance. Therefore, it is highly desirable to maneuver the capping ligand of noble metal nanocrystals according to the need of specific applications.

When one attempts to avoid the use of a specific capping ligand in synthesis, it turns out to be greatly difficult to find alternative ligands without affecting the morphology and yield of the nanocrystals. For example, Au nanorods (AuNRs) were exclusively synthesized by using CTAB or its analogs as the capping ligand [23–25]. No reports could be found in the literature on a robust synthesis of AuNRs with other capping ligands such as trisodium citrate (TSC). An alternative approach to solve this problem is to first synthesize the nanocrystals by adopting a well-established protocol in the literature, followed by a ligand exchange process to replace the native capping ligand with the desired one [19, 26–33]. Because the ligand exchange is usually driven by the difference in the binding strength of the capping ligands, it generally follows the rule of “the strong replaces the weak” and affords nanocrystals with increasingly strong capping ligands. Ligand exchange in the reverse order is particularly difficult, which imposes great limitations to the conventional ligand exchange processes in modulating the surface property of noble metal nanocrystals.

Toward a ligand exchange in the reverse order, recently, a successful case has been demonstrated by first growing a thin layer of Ag on AuNRs to remove the native capping ligand of cetyltrimethylammonium chloride (CTAC) and then etching Ag in the presence of TSC to obtain TSC-capped AuNRs [34]. Because this strategy relies on the epitaxial growth of Ag on Au nanocrystals, its extension to other metal nanocrystals needs to be further explored. Therefore, it is still highly desirable to find competent alternative strategies to achieve effective ligand exchange for flexibly tailored surface properties and thus broad applications of noble metal nanocrystals.

Herein, we report the use of diethylamine (DEA), whose binding affinity to the metal surface can be conveniently switched by controlling the pH, to serve as an intermediate ligand to enable the replacement of strong native capping ligands with weak ones. This strategy takes advantage of the switchable binding strength of DEA to the metal surface, depending on the pH: the lone electron pair of the N atom makes DEA a strong capping ligand, which enables a complete removal of the original capping ligand by its strong adsorption on the metal surface; while protonated by an acid, the DEA readily loses its capping ability, allowing its desorption from the metal surface and the adsorption of a designated capping ligand, even those very weak ones. Therefore, by using DEA as an intermediate capping ligand, the irreversible exchange order in the conventional ligand exchange processes could be overcome, making the capping ligand exchangeable by virtually any type of the ligands to afford custom surface properties. We demonstrate the efficacy of this strategy by on-demand exchange of capping ligands of cetyltrimethylammonium (CTA, positive), TSC (negative), PVP (neutral), and oleylamine (OAm, neutral). We believe this strategy is general and capable of producing noble metal nanocrystals with specific morphology and widely selected capping ligands, which paves the way to the applications of noble metal nanocrystals in many new regimes, as demonstrated in this work by the biological applications of AuNRs.

## 2. Results

### 2.1. Binding Energies of Capping Ligands on the Au Surface

**Figure 1:**
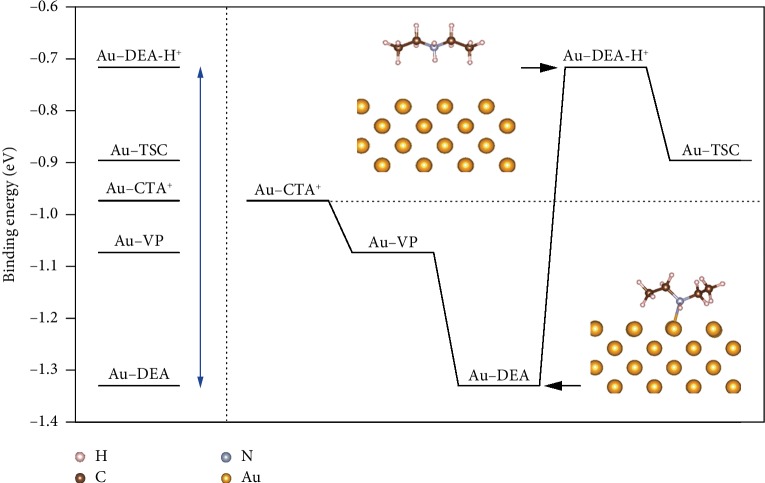
DFT calculations. Left: calculated binding energies (*E*_binding_) of different capping ligands adsorbed on the Au (110) surface. Right: a typical ligand exchange process based on the pH-tunable binding energy of DEA. Inset: optimized structures of DEA and DEA-H^+^ on the Au (110) surface.

Our strategy relies on the pH-switchable binding affinity of DEA on the metal surface to enable the replacement of strong capping ligands with weak ones. To validate this mechanism, we carried out density functional theory (DFT) calculations to evaluate the binding energies (*E*_binding_) of DEA in its original and protonated forms on the Au surface, in comparison with common capping ligands such as TSC, CTAB, and PVP. In this calculation, Au (110) was chosen as the adsorption surface, which exists on lateral faces of single-crystalline AuNRs [35]. The polymer of PVP was simplified as a monomer of *N*-vinylpyrrolidone (VP). The calculated binding energies, defined as *E*_binding_ = *E*_surface+adsorbate_ − (*E*_surface_ + *E*_adsorbate_), are summarized in Figure 1 (for more details, see Figures [Supplementary-material supplementary-material-1]–[Supplementary-material supplementary-material-1] and [Supplementary-material supplementary-material-1]).

As shown in Figure 1 (left), *E*_binding_ increases in the order of Au–DEA-H^+^ (−0.72 eV) < Au–TSC (−0.90 eV) < Au–CTA (−0.97 eV) < Au–VP (−1.07 eV) < Au–DEA (−1.33 eV). Therefore, the ligand exchange can proceed only in the direction of TSC → CTAB → PVP, driven by the difference in the binding energy. Interestingly, the binding energies of DEA in its original and protonated forms well envelop those of TSC, CTAB, and PVP. DEA in its original form shows the highest binding energy among all ligands investigated, which can be attributed to the formation of strong Au–N bonds on the Au surface, as indicated by the optimized structure (Figure 1, inset). It facilitates the efficient removal of many common capping ligands from the surface of noble metal nanocrystals. After protonation, the DEA-H^+^ becomes the weakest ligand among all ligands investigated, due to the breaking of the Au–N bonds (Figure 1, inset). It makes the subsequent ligand exchange favorable with many common ligands, including those very weak ones such as TSC. Therefore, by employing DEA as an intermediate ligand, the irreversible exchange order in conventional ligand exchange strategies could be overcome from the perspective of energetics.

We take the ligand exchange from CTAB to TSC for example and demonstrate how “the strong replaces the weak” rule can be overcome by our strategy (Figure 1, right). Because Au nanocrystals with the capping ligands of CTAB and DEA carry opposite surface charges (discussed below), a direct addition of DEA to the colloid of CTAB-capped Au nanocrystals will cause irreversible aggregation due to the neutralization of the surface charges. To avoid it, the surface ligand of CTAB is first replaced with PVP prior to the addition of DEA. This ligand exchange occurs spontaneously because PVP binds much more strongly to the Au surface than CTAB, giving rise to Au nanocrystals with electrostatically neutral surfaces and well-retained colloidal dispersion thanks to the steric hindrance of the polymeric PVP. Because the binding energy of PVP on the Au surface is still much weaker than that of DEA, DEA-capped Au nanocrystals can be readily obtained by a further ligand exchange. The ligand of DEA is then protonated in the presence of TSC, leading to the detachment of DEA and adsorption of TSC despite its low adsorption energy on the Au surface. By this means, “the strong replaces the weak” rule in conventional ligand exchange processes can be successfully overcome to afford nanocrystals with weak capping ligands.

### 2.2. Experimental Ligand Exchange of AuNRs to Replace the Native CTAB with TSC

**Figure 2:**
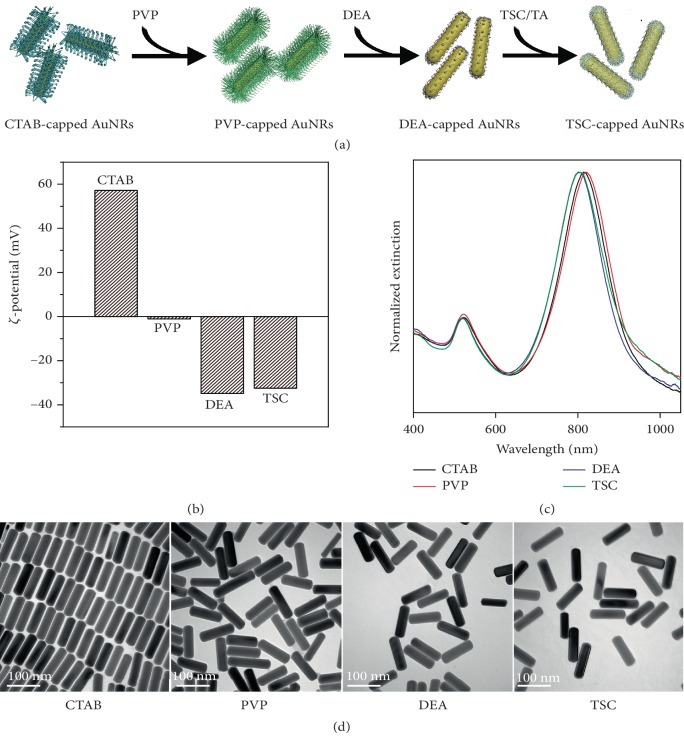
A typical ligand exchange of AuNRs to replace the native CTAB with TSC. (a) A scheme illustrating the ligand exchange process. (b, c) *ζ*-potentials and UV-vis-NIR spectra of the AuNRs obtained at different stages of the ligand exchange. (d) TEM images of the AuNRs with different capping ligands.

To verify the ligand exchange experimentally, colloidal AuNRs were synthesized by following a typical procedure in the literature [36]. The native capping ligand of CTAB was replaced with a weak and biocompatible capping ligand of TSC, making these AuNRs suitable for biological applications (Figure 2(a)).

First, the native capping ligand of CTAB on the AuNRs was changed to PVP by mixing the colloid with PVP in an ethanolic solution. During this process, the *ζ*-potential of the AuNRs decreased from +57.2 to ~0 mV (measured value, −1.1 mV), indicating a successful ligand exchange (Figure 2(b)). The measured value of −1.1 mV may arise from a fluctuation of the *ζ*-potential values around 0 due to the electrostatic neutrality of the nanorod surface. The UV-vis-near-infrared (NIR) spectrum of the PVP-capped AuNRs remained almost identical to that of the AuNRs before the ligand exchange (Figure 2(c)). A slight redshift of the surface plasmon resonance (SPR) band could be attributed to a change in the surface ligand and thus the dielectric environment of the AuNRs [37]. Then, a ligand exchange occurs spontaneously when DEA was introduced into the colloid of the PVP-capped AuNRs. A dramatic change in the *ζ*-potential from ~0 to −34.9 mV could be observed (Figure 2(b)). We hypothesize that DEA loses a proton from N–H when it adsorbs onto the Au surface, which accounts for the negative charges of the AuNRs to ensure the colloidal dispersity of the AuNRs. In fact, the N–H bond in the DEA molecule is so active that the corresponding ^1^H NMR signal could be hardly detected when it is dissolved in D_2_O (discussed later). The ligand exchange also leads to a significant blueshift of the SPR band due to the change of the capping ligand on the AuNRs (Figure 2(c)). Finally, the DEA molecules on the surface of the AuNRs were protonated by an acid, for example, tannic acid (TA), in the presence of a designated capping ligand of TSC. It produced TSC-capped AuNRs, with the *ζ*-potential measured to be −32.5 mV (Figure 2(b)). The UV-vis-NIR spectrum of the AuNRs remained almost unchanged, which ruled out the aggregation of the AuNRs in this process (Figure 2(c)).

Therefore, the capping ligand on the AuNRs has been successfully exchanged from the native CTAB to TSC by employing DEA as an intermediate ligand and taking advantage of its pH-switchable binding affinity to the metal surface. No changes in the shape and size of the AuNRs were discernible by the transmission electron microscopy (TEM) imaging (Figure 2(d)), which could be attributed to the mild conditions that have been involved in the whole ligand exchange process. It also confirms the absence of agglomerations of the AuNRs in all colloids examined, which is consistent with the dynamic light scattering (DLS) results ([Supplementary-material supplementary-material-1]). The well-retained colloidal dispersity of the AuNRs could be attributed to the steric hindrance when PVP is adsorbed on the surface and strong electrostatic repulsion force when DEA or TSC is adsorbed on the surface (absolute values of *ζ*-potentials, >30 mV as discussed above).

### 2.3. The Effectiveness of the Ligand Exchange

**Figure 3:**
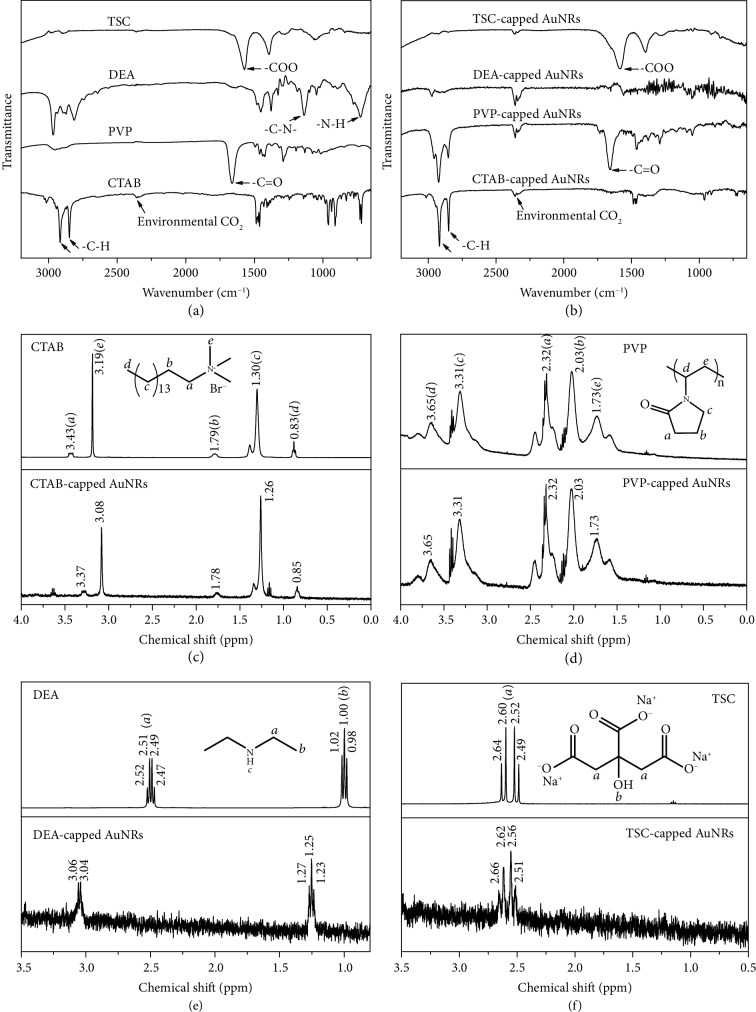
Spectral evidence on the effectiveness of the ligand exchange. (a) FTIR spectra of pure capping ligands including CTAB, PVP, DEA, and TSC. (b) FTIR spectra of AuNRs with different capping ligands obtained at different stages of the ligand exchange. (c–f) ^1^H NMR spectra of the AuNRs with the capping ligands of CTAB, PVP, DEA, and TSC, respectively. The spectra of pure chemicals are listed for comparison.

To unambiguously reveal the evolution of the capping ligand during the ligand exchange, the AuNRs were subjected to the Fourier transform infrared (FTIR) and nuclear magnetic resonance (NMR) spectroscopies (Figure 3). In particular, the AuNRs were thoroughly washed before the NMR analysis so that the signals were from the surface-bound capping ligands rather than free molecules. The FTIR spectrum of the CTAB-capped AuNRs shows prominent vibrational peaks at 2854 and 2925 cm^−1^, corresponding to the –CH_2_– groups of the CTAB molecules (Figures 3(a) and 3(b)). The ^1^H NMR spectrum of the AuNRs shows characteristic peaks of CTAB with negligible shifts relative to those of the pure chemical, confirming the presence of native CTAB on the Au surface (Figure 3(c)). After the ligand exchange with PVP, while the typical peaks have been retained in the FTIR spectrum (PVP also contains –CH_2_– groups), a well-resolved peak emerges at 1654 cm^−1^ corresponding to the –C=O groups of the amide, indicating successful adsorption of PVP on the AuNRs (Figures 3(a) and 3(b)). The ^1^H NMR spectrum of the AuNRs shows well-resolved peaks that perfectly match those of the pure chemical of PVP (Figure 3(d)). The original peaks from CTAB disappear completely, which confirms the full desorption of CTAB from the Au surface. All these results validate a successful ligand exchange of the AuNRs from native CTAB to PVP.

A ligand exchange process was further carried out to replace PVP with DEA on the surface of the AuNRs. From the FTIR, the peak corresponding to the –C=O groups of PVP disappears, which is a clear indication of the complete desorption of PVP from the Au surface (Figures 3(a) and 3(b)). As the FTIR spectrum becomes featureless, the presence of DEA on the Au surface could not be revealed in this spectrum, which, however, could be verified by the NMR (Figure 3(e)). The ^1^H NMR peaks of the AuNRs at chemical shifts of ~1.0 and ~2.5 ppm correspond to the H atoms in the –CH_3_ and –CH_2_– groups of DEA, respectively. No signals from H in the –NH– group could be detected from both the DEA-capped AuNRs and the pure chemical of DEA, indicating that these H atoms are highly active and easily deprotonated. Compared with the free DEA, the ^1^H NMR peaks of the DEA adsorbed on the AuNRs undergo a significant downfield shift and line broadening. The downfield shift indicates an effective DEA → Au electron transfer, which leads to a significant decrease in the electron density around the H atoms, and the line broadening can be attributed to the attenuated molecular tumbling of DEA on the AuNRs and hence the reintroduction of dipole-dipole spin interactions [38–41]. Both observations are consistent with the DFT calculations: while CTAB, PVP, and TSC bind weakly to the Au surface without forming covalent bonds, the DEA molecules attach to the Au surface via the formation of strong covalent Au–N bonds (Figures 1 and [Supplementary-material supplementary-material-1]–[Supplementary-material supplementary-material-1]). The high binding energy of DEA on the AuNRs accounts for the line broadening of its ^1^H NMR signals. Moreover, the resonances of the PVP disappear from the ^1^H NMR spectrum, which again confirms the complete desorption of the prior capping ligand of PVP and verifies the effective ligand exchange.

Eventually, the DEA on the AuNRs was replaced with the designated capping ligand of TSC. After the ligand exchange, the FTIR spectrum of the AuNRs shows typical vibrational peaks of TSC (Figures 3(a) and 3(b)). For example, the peak at 1584 cm^−1^ can be ascribed to the carboxylate groups of TSC. The ^1^H NMR spectrum of the AuNRs shows characteristic peaks at ~2.6 ppm, corresponding to the –CH_2_– groups of TSC (Figure 3(f)). The peaks at ~1.25 and ~3.0 ppm disappear, which suggests the absence of DEA from the Au surface. All these observations confirm a successful replacement of DEA with TSC on the surface of AuNRs. It is worth noting that no other signals, for example, those from tannic acid, could be found in the FTIR or ^1^H NMR spectrum of the TSC-capped AuNRs (Figures 3(b) and 3(d) and [Supplementary-material supplementary-material-1]). It hints that tannic acid, which was used to protonate DEA to initiate the ligand exchange, is absent from the Au surface, and hence, TSC is the only capping ligand on the AuNRs. Therefore, by combining the FTIR and NMR analyses, the evolution of the capping ligands on the AuNRs and thus the effectiveness of the ligand exchange strategy have been unambiguously verified.

### 2.4. Broad Applicability of the Ligand Exchange Strategy

**Figure 4:**
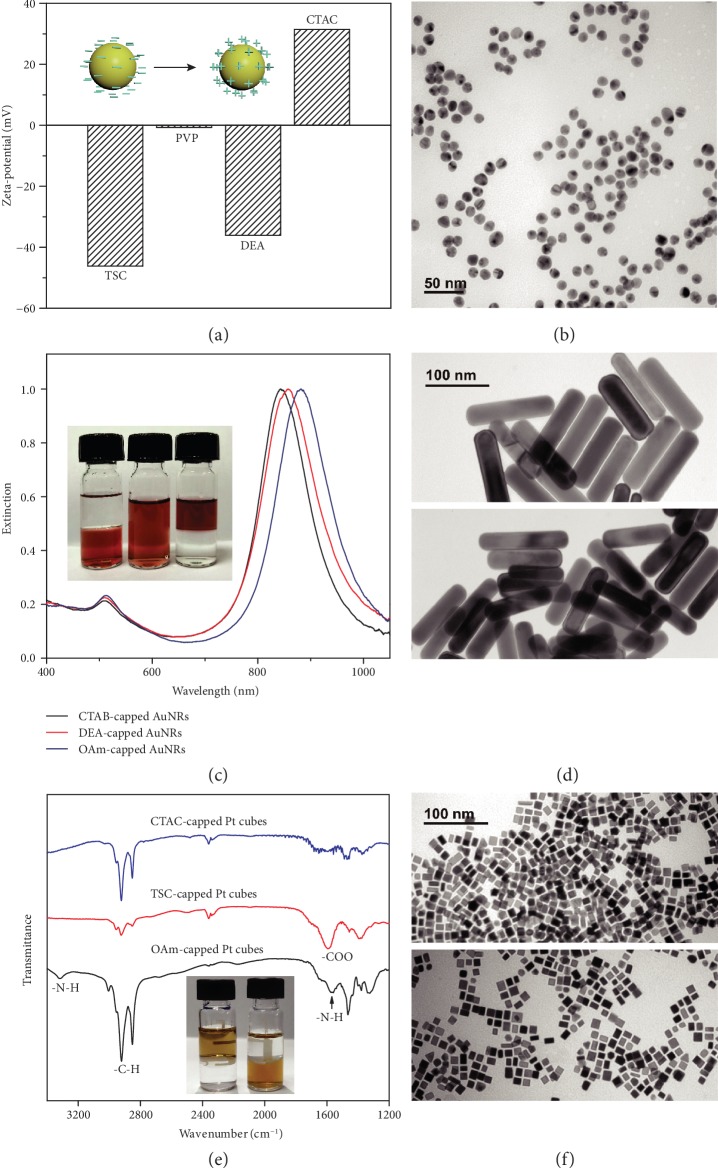
Applicability of the ligand exchange strategy. (a, b) Ligand exchange of AuNSs to replace native TSC with CTAC: (a) *ζ*-potentials of the AuNSs obtained at different stages of the ligand exchange and (b) TEM image of the CTAC-capped AuNSs. (c, d) Ligand exchange of AuNRs to replace native CTAB with OAm: (c) UV-vis-NIR spectra of the AuNRs with native CTAB, DEA, and OAm, respectively, and (d) TEM images of the AuNRs with the capping ligands of native CTAB (top) and OAm (bottom). The inset in (c) shows the photographs of, from left to right, CTAB-capped AuNRs in H_2_O, DEA-capped AuNRs in ethanol, and OAm-capped AuNRs in 1-octanol (bottom phase: H_2_O). (e, f) Ligand exchange of Pt nanocubes to replace native OAm with CTAC and TSC: (e) FTIR spectra of the Pt nanocubes with the capping ligands of OAm, CTAC, and TSC, respectively, and (f) TEM images of the Pt nanocubes with the capping ligands of CTAC (top) and TSC (bottom). The inset in (e) shows the photographs of (from left to right) OAm-capped Pt nanocubes in toluene (bottom phase: H_2_O) and TSC-capped Pt nanocubes in H_2_O (upper phase: toluene).

Our ligand exchange strategy by employing DEA as the intermediate ligand is general and broadly applicable in different ligand exchange scenarios. To demonstrate it, we show that the TSC-capped Au nanospheres (AuNSs) obtained from a traditional citrate reduction synthesis could be conveniently converted to CTA-capped ones (Figures 4(a) and 4(b)), which are in the reverse order of the ligand exchange as discussed above. Because the TSC-capped AuNSs would undergo irreversible aggregation upon a direct addition of DEA, we first introduced PVP into the colloid of the AuNSs, giving rise to PVP-capped AuNSs. The *ζ*-potential of the AuNSs changed from −46.2 mV to ~0 (measured value, −0.68 mV), which confirmed the adsorption of the electrostatically neutral polymer of PVP in place of the negatively charged capping ligand of TSC on the Au surface (Figure 4(a)). The PVP was then replaced with DEA, giving rise to negatively charged AuNSs with a *ζ*-potential of −36.1 mV. By further protonating DEA with HCl in the presence of CTAC, CTAC-capped AuNSs were eventually obtained as the final product. The *ζ*-potential of the AuNSs increased to +31.5 V. The *ζ*-potential well exceeds 30 mV, indicating high colloidal dispersity of the AuNSs, which can be further verified by TEM imaging and UV-vis-NIR spectroscopy (Figures 4(b) and [Supplementary-material supplementary-material-1]).

The wide applicability of the ligand exchange strategy could be also evidenced by converting aqueous noble metal nanocrystals into oil-soluble ones with nonionic, electrostatically neutral capping ligands. To demonstrate it, we successfully changed the capping ligand of AuNRs from native CTAB to OAm with a phase transfer from water to 1-octanol (Figures 4(c) and 4(d)). Synthetically, the capping ligand on the AuNRs was exchanged from the native CTAB to PVP and DEA in sequence. To facilitate the further ligand exchange with OAm, the DEA-capped AuNRs were dissolved in ethanol, because ethanol is a solvent for both DEA and OAm. By protonating the DEA-capped AuNRs with acetic acid in the presence of OAm, OAm-capped AuNRs were readily obtained, which were then transferred to 1-octanol, forming a stable colloid (Figure 4(c), inset). The UV-vis-NIR spectrum of the OAm-capped AuNRs showed prominent SPR bands that were similar to those obtained before the ligand exchange (Figure 4(c)). Neither broadening of the bands nor the emergence of new extinction bands could be observed, which confirms the colloidal dispersity of the AuNRs. A significant redshift of the dipole-mode SPR band could be attributed to the different capping ligands and solvents and thus the different dielectric environments of the AuNRs. The TEM images showed essentially the same morphology, indicating the absence of aggregations during the ligand exchange process (Figure 4(d)).

In a previous report, we already showed a conversion of OAm-capped noble metal nanocrystals into DEA-capped ones [19]. By using the pH-switchable binding affinity of DEA on the metal surface, we demonstrate that these DEA-capped nanocrystals, for example, Pt nanocubes, could be further converted to CTAC- and TSC-capped nanocrystals (Figures 4(e) and 4(f)). As a result, Pt nanocubes could be transferred from an oil phase to an aqueous phase (Figure 4(e), inset). The effectiveness of the ligand exchange could be confirmed by FTIR spectroscopy, which showed characteristic vibrational signals corresponding to the ligands of CTAC and TSC, respectively (Figure 4(e)). The TEM images confirmed that the colloidal dispersity of the Pt nanocubes was well retained after they were transferred to the aqueous phase (Figure 4(f)).

**Figure 5:**
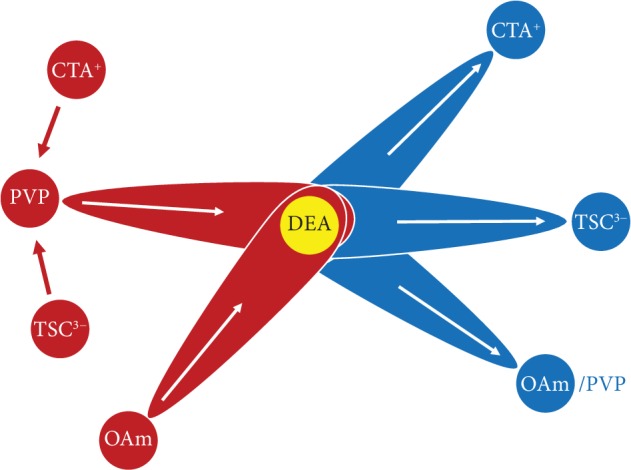
A diagram for the ligand exchange strategy. By using DEA as an intermediate capping ligand (“pivot”), different types of capping ligands could be mutually exchanged on the surface of noble metal nanocrystals.

All the above demonstrations (for additional results on the ligand exchange of Ag nanocrystals, see [Supplementary-material supplementary-material-1]) verify the general applicability of the ligand exchange strategy (Figure 5). Here, DEA serves as an intermediate capping ligand, which plays a “pivotal” role in the ligand exchange process. It enables mutual exchange of common capping ligands on noble metal nanocrystals to afford tailored surface properties, which paves a way to their broad applications in different fields.

### 2.5. Biotoxicity and Photothermal Effect of AuNRs with Different Capping Ligands

**Figure 6:**
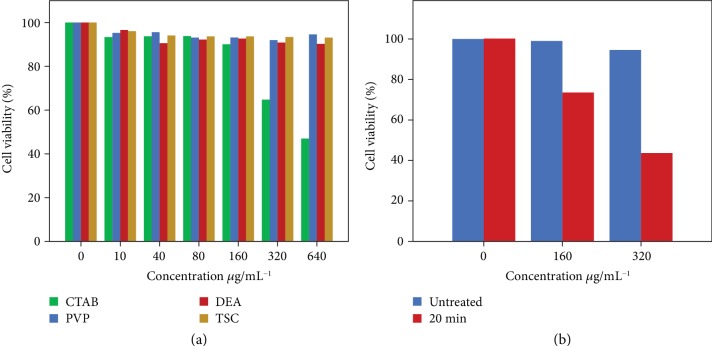
Biological application of the AuNRs with different capping ligands. (a) Biotoxicity of the AuNRs with the capping ligand of CTAB, PVP, DEA, and TSC, respectively, at different concentrations, showing the viability of the cells after incubation with the AuNRs for 3 days. (b) Viability of the cells after photothermal treatment with TSC-capped AuNRs of different concentrations.

We take AuNRs as an example and demonstrate the application of noble metal nanocrystals with tailored capping ligands (Figure 6). As discussed above, conventionally, the synthesis of AuNRs was most successful by employing CTAB or its analog as the capping ligand. By the ligand exchange, the native CTAB on the surface of the AuNRs could be readily replaced with PVP, DEA, and eventual TSC. The cytotoxicity of the AuNRs with different capping ligands was evaluated on mouse fibroblast L929 proliferation. Typically, the cells were incubated with the respective AuNRs at different concentrations in the range of 20–1000 *μ*g mL^−1^ for 1 day ([Supplementary-material supplementary-material-1]) and 3 days (Figure 6a). Cell viability was evaluated by the alamarBlue assay and with a LIVE/DEAD viability/cytotoxicity kit ([Supplementary-material supplementary-material-1]). It is clear that the cytotoxicity of CTAB-capped AuNRs increased with their concentrations. At a concentration of 640 *μ*g mL^−1^, the cell viability decreased to 67.1% on the first day and 46.9% on the third day, which suggests strong cytotoxicity of the CTAB-capped AuNRs at this concentration. Compared with the CTAB-capped AuNRs, the AuNRs with the capping ligands of PVP, DEA, and TSC showed much lower cytotoxicity. Of these three types of AuNRs, there was no significant difference in the cytotoxicity when the concentration of the AuNRs was lower than 640 *μ*g mL^−1^. In particular, with the TSC-capped AuNRs (concentration, 640 *μ*g mL^−1^), the cell viability was 89.4% on the first day and 93.1% on the third day, which was much higher than that with the CTAB-capped AuNRs. In the LIVE/DEAD-stained fluorescent images ([Supplementary-material supplementary-material-1]), dominant live cells (green color) were observed in all the three groups (PVP-, DEA-, and TSC-capped AuNRs) when the concentration of the AuNRs was lower than 640 *μ*g mL^−1^. For comparison, a large number of dead cells (red color) could be observed when the cells were incubated with CTAB-capped AuNRs. These results confirmed the significantly enhanced biocompatibility of the AuNRs when the native capping ligand of CTAB was replaced with PVP, DEA, and TSC. It is worth noting that with another type of cells, *i.e.*, human malignant melanoma cell line A375, the TSC-capped AuNRs showed similar cytotoxicity at different concentrations, which indicates that the cytotoxicity of the TSC-capped AuNRs was general and not specific to certain types of the cells (Figures [Supplementary-material supplementary-material-1] and [Supplementary-material supplementary-material-1]).

The high biocompatibility of the TSC-capped AuNRs renders them highly applicable in biological applications. For example, these AuNRs are promising in hyperthermia therapy of tumors by taking advantage of their light absorption in the near-infrared range of the spectrum. As a preliminary demonstration, A375 cells were incubated with the TSC-capped AuNRs. Under the laser radiation (wavelength: 808 nm) for 20 min, a significant loss of the cell viability has been observed (Figure 6(b)). The cell viability decreases with an increasing concentration of the AuNRs. In clear contrast, no significant decrease in the viability of the cells could be observed in the absence of the laser radiation, thanks to the low cytotoxicity of the TSC-capped AuNRs. We believe these biocompatible AuNRs may be useful in a much broader range of biological applications, such as biosensing, labeling, and imaging.

## 3. Discussion

In summary, we have proposed a general ligand exchange strategy for customizing the surface property of noble metal nanocrystals by using DEA as an intermediate ligand. The binding affinity of DEA to the surface of metal nanocrystals is pH-switchable, which allows it to first replace the native ligand and then be replaced with virtually any type of desired capping ligands, affording tunable surface properties. The significance is that it overcomes the irreversibility of the conventional ligand exchange processes and allows the native ligand to be exchanged by weaker capping ligands. Thus, the limitation of “the strong replaces the weak” in the conventional ligand exchange strategies has been greatly circumvented, and ligands with different binding affinities can now be introduced to the surface of the metal nanocrystals according to the need of specific applications. In this work, the effectiveness of this strategy has been verified with nanocrystals of Au (Figures 1–3 and 4(a)–4(d)), Pt (Figures 4(e) and 4(f)), and Ag ([Supplementary-material supplementary-material-1]). Its effectiveness in the ligand exchange of many other noble metal nanocrystals is still under investigation. We believe this ligand exchange strategy is general and extendable to a broad range of noble metal nanocrystals to achieve custom surface properties, which may greatly expand the scope of their potential applications.

## 4. Materials and Methods

### 4.1. Synthesis of CTAB-Capped AuNRs

CTAB-capped AuNRs were synthesized according to a previous report [36]. A seed solution was first prepared by injecting 1 mL of NaBH_4_ (6 mM, freshly prepared) into 10 mL of a solution containing 0.25 mM HAuCl_4_ and 0.1 M CTAB. After rapid inversions for 2 min, the seed solution was kept at room temperature for 30 min before use. Another solution was prepared by mixing 11.2 g of CTAB and 1.974 g of sodium oleate in 400 mL of H_2_O at 55°C until it becomes transparent. After this solution was transferred to a water bath at 30°C, 38.4 mL of AgNO_3_ (4 mM) was added to the solution, and the solution stayed static for 15 min. Then, 400 mL of HAuCl_4_ (1 mM) was added, followed by slow stirring for 90 min until it became colorless. Next, 3.36 mL of HCl (12.1 M) was added to this solution, and the solution was stirred for another 15 min. After the addition of 6.4 mL of ascorbic acid (0.1 M), 640 *μ*L of the seed solution was quickly injected into the growth solution. The solution was gently mixed for 30 s and left undisturbed at 28°C for 12 h. The AuNRs were collected by centrifugation and redispersed in 80 mL of H_2_O (Au: ~5 mM).

### 4.2. Ligand Exchange of AuNRs to Replace the Native CTAB with TSC

(1) The CTAB-capped AuNRs (10 mL, containing ~5 mM Au) were centrifuged to remove excess CTAB and redispersed in 10 mL of H_2_O. Then, 10 mL of the AuNRs was mixed with 30 mL of ethanol and 1 mL of PVP (Mw 10,000, 50 mg mL^−1^) and stirred overnight. The resulting PVP-capped AuNRs were centrifuged, washed with water, and redispersed in 10 mL of H_2_O. (2) The PVP adsorbed on the AuNRs was replaced with DEA. Typically, 500 *μ*L of DEA was added to 10 mL of the PVP-capped AuNRs, and the solution was stirred for 2 h. This process was then repeated to ensure the complete ligand exchange. The DEA-capped AuNRs were collected by centrifugation and redispersed in 10 mL of H_2_O. (3) The DEA adsorbed on the AuNRs was replaced with TSC. Typically, 200 *μ*L of TSC (10 mg mL^−1^) and 400 *μ*L of tannic acid (10 mg mL^−1^) were added to the aqueous solution of DEA-capped AuNRs. After stirring for 1 h, the TSC-capped AuNRs were collected by centrifugation and redispersed in 10 mL of H_2_O.

### 4.3. Ligand Exchange of AuNRs to Replace the Native CTAB with OAm

Steps (1) to (2) of the ligand exchange were similar to the procedures as described above, except that the DEA-capped AuNRs were redispersed in 20 mL of ethanol. In step (3), the DEA adsorbed on the AuNRs was replaced with OAm. Typically, 800 *μ*L of OAm and 200 *μ*L of acetic acid were added to the above ethanolic solution. After stirring for 25 min, the OAm-capped AuNRs were collected by centrifugation and redispersed in 20 mL of 1-octanol.

### 4.4. Cytotoxicity Tests of the AuNRs with Different Capping Ligands

Human malignant melanoma cell line A375 and mouse fibroblast L929 were obtained from the Stem Cell Bank, Chinese Academy of Sciences, and incubated in a humidified incubator with 5% CO_2_ at 37°C. The complete growth medium for A375 was Dulbecco's modified Eagle's medium (DMEM, Gibco) supplemented with 10% fetal bovine serum (FBS, Biological Industries), 100 units mL^−1^ penicillin (HyClone), and 100 *μ*g mL^−1^ streptomycin (HyClone). The complete growth medium for L929 was DMEM supplemented with 10% horse serum (HS, Gibco), 100 units mL^−1^ penicillin, and 100 *μ*g mL^−1^ streptomycin. To investigate the cytotoxicity of the AuNRs, L929 fibroblasts and A375 cells were seeded, respectively, in a 96-well plate (Costar) at a density of 5000 cells per well and incubated with AuNRs at different concentrations in the range of 20–1000 *μ*g mL^−1^. Cell viability was evaluated by the alamarBlue assay (Molecular Probes). Fluorescence was read at 560/600 nm with a microplate reader (Molecular Devices). The cells were incubated for 1 and 3 days and tested, respectively. Tests were repeated six times for each group. The LIVE/DEAD viability/cytotoxicity (Invitrogen) assay was also applied to evaluate the cytotoxicity of AuNRs. After being cultured for 1 and 3 days, the cells were washed with DPBS and cultured with a LIVE/DEAD staining reagent for 45 min protected from light in the incubator. The cells were observed under the inverted fluorescence microscope (IX53, Olympus).

### 4.5. Evaluation of the Photothermal Effect of the AuNRs

A375 cells were seeded in a 96-well plate at a density of 10000 cells per well. After cell adhesion, the medium was changed to complete growth medium containing 160 or 320 *μ*g mL^−1^ AuNRs for the following incubation for 24 h. The cells were irradiated with a laser (808 nm, 1.25 W cm^−2^, 20 min) and tested with alamarBlue.

### 4.6. DFT Calculations

Electronic structure calculations were performed within the density functional theory (DFT) at the level of Perdew, Burke, and Ernzerhof (PBE) functional [42, 43], as implemented in the Vienna Ab initio Simulation Package (VASP) [44–46], a plane-wave pseudopotential package. The plane-wave cutoff energy was set to 400 eV, and the first-order Methfessel-Paxton scheme was used with a smearing width of 0.2 eV. Dipole corrections were applied along the *z*-axis. The PBE-D3 method was employed to correct van der Waals interaction [47]. The convergence criterion of the electronic self-consistent loop was 1 × 10^−5^ eV in energy differences for solving the electronic wave function. All geometries (atomic coordinates) were considered converge if forces are smaller than 1 × 10^−3^ eV Å^−1^. The Au electrode consists of a 4 × 4 Au (110) surface with 4 layers. We fixed the bottom two layers using the experimental lattice parameter (4.07 Å). The simulation box is 26 Å along the *z*-axis with a vacuum of 15 Å and kept fixed during the calculation. We considered five adsorbates in our calculation, including CTA^+^, VP, DEA, DEA-H^+^, and TSC^3−^. The spin polarization effect was taken into consideration. The solvation effect has been taken into consideration with an implicit solvation model that describes the effect of electrostatics, cavitation, and dispersion on the interaction between a solute and solvent as implemented in VASPsol [48, 49]. For the charged systems, we simulated CTA^+^ and DEA-H^+^ by removing one electron from neutral molecules. We simulated TSC^3−^ by adding three electrons to the neutral molecule. No explicit cations were involved in the simulations.

## Supplementary Materials

Supplementary Materials Materials and Methods. Figure S1: optimized structure of CTA adsorbed on the Au (110) surface. Figure S2: optimized structure of VP (as a simplified model of PVP) adsorbed on the Au (110) surface. Figure S3: optimized structures of DEA and DEA-H^+^ adsorbed on the Au (110) surface. Figure S4: optimized structure of TSC adsorbed on the Au (110) surface. Figure S5: hydrodynamic sizes of the AuNRs obtained at different stages of the ligand exchange, measured by dynamic light scattering (DLS). Figure S6: FTIR spectra of TSC and tannic acid (TA). Figure S7: ligand exchange of AuNSs to replace the native TSC with CTAC. Figure S8: applicability of the ligand exchange strategy to Ag nanospheres. Figure S9: biotoxicity of the AuNRs with different capping ligands and concentrations. Figure S10: fluorescent images showing the LIVE/DEAD staining of L929 cells after incubation with AuNRs with different capping ligands and concentrations for 3 days. Figure S11: biotoxicity of the TSC-capped AuNRs at different concentrations. Figure S12: fluorescent images showing the LIVE/DEAD staining of A375 cells after incubation with TSC-capped AuNRs of different concentrations for 3 days. Table S1: detailed binding energies of CTA^+^, VP, DEA, DEA-H^+^, and TSC adsorbed on Au (110) surfaces.
