# Supplementary material for: Customizable Ligand Exchange for Tailored Surface Property of Noble Metal Nanocrystals
Source: Research (Wash D C). 2020 Jan 21;2020:2131806. doi: 10.34133/2020/2131806 (PMC6998038; doi:10.34133/2020/2131806)
Supplement: Supplementary Materials — Materials and Methods. Figure S1: optimized structure of CTA adsorbed on the Au (110) surface. Figure S2: optimized structure of VP (as a simplified model of PVP) adsorbed on the Au (110) surface. Figure S3: optimized structures of DEA and DEA-H+ adsorbed on the Au (110) surface. Figure S4: optimized structure of TSC adsorbed on the Au (110) surface. Figure S5: hydrodynamic sizes of the AuNRs obtained at different stages of the ligand exchange, measured by dynamic light scattering (DLS). Figure S6: FTIR spectra of TSC and tannic acid (TA). Figure S7: ligand exchange of AuNSs to replace the native TSC with CTAC. Figure S8: applicability of the ligand exchange strategy to Ag nanospheres. Figure S9: biotoxicity of the AuNRs with different capping ligands and concentrations. Figure S10: fluorescent images showing the LIVE/DEAD staining of L929 cells after incubation with AuNRs with different capping ligands and concentrations for 3 days. Figure S11: biotoxicity of the TSC-capped AuNRs at different concentrations. Figure S12: fluorescent images showing the LIVE/DEAD staining of A375 cells after incubation with TSC-capped AuNRs of different concentrations for 3 days. Table S1: detailed binding energies of CTA+, VP, DEA, DEA-H+, and TSC adsorbed on Au (110) surfaces. [file 2131806.f1.docx]

Supplementary Information

Customizable Ligand Exchange for Tailored Surface Property

of Noble Metal Nanocrystals

Qikui Fan,^1†^ Hao Yang,^2†^ Juan Ge,^1^ Shumeng Zhang,^1^ Zhaojun Liu,^1^ Bo Lei,^1^

Tao Cheng,^2^ Youyong Li,^2^ Yadong Yin,^3^ and Chuanbo Gao^1^*

*^1^Frontier Institute of Science and Technology, and State Key Laboratory of Multiphase Flow in Power Engineering, Xi’an Jiaotong University, Xi’an, Shaanxi 710054, China.*

*^2^Institute of Functional Nano & Soft Materials (FUNSOM), Jiangsu Key Laboratory for Carbon-Based Functional Materials & Devices, Joint International Research Laboratory of Carbon-Based Functional Materials and Devices, Soochow University, Suzhou, Jiangsu 215123, China.*

*^3^Department of Chemistry, University of California, Riverside, California 92521, USA.*

*^†^These authors contributed equally to this work.*

**Correspondence should be addressed to Chuanbo Gao; gaochuanbo@mail.xjtu.edu.cn*

**Materials and Methods**

*Synthesis of TSC-capped Au nanospheres (AuNSs).* TSC-capped AuNSs were synthesized by a traditional Frens’s method.^1^ Typically, 95 µL of HAuCl_4_ (0.25 M) was injected into a flask containing 47.5 mL of H_2_O. After the solution was heated to its boiling point (~ 100 °C), 2.5 mL of TSC (10 mg/mL) was quickly injected into the solution. The solution was heated until its color changed from yellow to wine-red. The resulting sol of the AuNSs was collected as a stock solution (Au: ~ 0.475 mM).

*Synthesis of OAm-capped Pt nanocubes (PtNCs).* OAm-capped PtNCs were synthesized by following a reported protocol with modification.^2^ Typically, in a round-bottom flask, 20 mL of OAm was pre-heated in CO at 155 °C. Then, 800 µL of H_2_PtCl_6_ (50 mM) was injected into the solution. The reaction was allowed to proceed at 155 °C for 35 min. The resulting PtNCs were collected by centrifugation, washed with toluene, and redispersed in 5 mL of toluene (Pt: ~ 8 mM).

*Ligand exchange of AuNSs to replace the native TSC by CTAC.* (1) In a typical ligand exchange, 10 mL of the TSC-capped AuNSs (Au: ~ 0.475 mM) was mixed with 30 mL of ethanol and 500 µL of PVP (Mw 10,000, 50 mg mL^–1^). After stirring overnight, PVP-capped AuNSs were collected by centrifugation and redispersed in 10 mL of H_2_O. (2) The PVP adsorbed on the AuNSs was replaced by DEA. Typically, 500 µL of DEA was added into 10 mL of the PVP-capped AuNSs and stirred for 2 h. This process was repeated for another time for complete ligand exchange. The DEA-capped AuNSs were collected by centrifugation and redispersed in 5 mL of H_2_O. (3) The DEA adsorbed on the AuNSs was replaced by CTAC. Typically, 400 µL of CTAC (0.1 M) and 50 µL of HCl (0.12 M) were added into 5 mL of the DEA-capped AuNSs and the solution was stirred for 10 min. The CTAC-capped AuNSs were collected by centrifugation and redispersed in 5 mL of H_2_O.

*Ligand-exchange of PtNCs to replace the native OAm by TSC.* (1) In a typical ligand-exchange, 2 mL of OAm-capped PtNCs (Pt, ~ 8 mM) were washed with ethanol and redispersed in 2 mL of ethanol. Then, 300 µL of DEA was added to the solution, which was kept static at room temperature for 24 h. The PtNCs were then collected by centrifugation, washed with ethanol/water, and redispersed in 2 mL of H_2_O. (2) To obtain TSC-capped PtNCs, 1 mL of the DEA-capped PtNCs was mixed with 50 µL of TSC (10 mg mL^–1^) and 50 µL of tannin acid (10 mg mL^–1^) and stirred for 20 min. The TSC-capped PtNCs were collected by centrifugation and redispersed in 1 mL of H_2_O. (3) To obtain CTAC-capped PtNCs, 1 mL of the DEA-capped PtNCs was mixed with 50 µL of CTAC (0.1M) and 50 µL of HCl (0.01M) and stirred for 20 min. The CTAC-capped PtNCs were collected by centrifugation and redispersed in 1 mL of H_2_O.

*Material characterizations.* (1) Transmission electron microscopy (TEM) was performed on an HT7700 operated at 100 kV or a JEOL JEM-F200 (HR) operated at 200 kV. The AuNRs and PtNCs with the capping ligands of CTAB, PVP, DEA, or TSC were centrifuged, washed with H_2_O, and redispersed in H_2_O to form a homogeneous sol. Then, 10 µL of the sol was dropped onto a carbon-supported copper grid and dried in air at room temperature. The metal nanocrystals on the grids were then subjected to the TEM imaging. (2) Dynamic light scattering (DLS) and ζ-potential analysis were performed on a Beckman Coulter Delsa Nano C particle analyzer. (3) UV-vis-near IR (NIR) spectra (λ: 300–1000 nm) were measured on an Ocean Optics HR2000+ES UV-vis-NIR spectrophotometer with a DH-2000-Bal light source. (4) Fourier-transform infrared (FTIR) spectra were measured on a Nicolet 6700 FT-IR spectrometer using the attenuated total reflectance (ATR) mode with a Smart iTR accessary. The FTIR spectra of pure chemicals of TSC, PVP, DEA, and CTAB were recorded by placing the pure chemicals (in the form of a powder or a liquid) onto the sample stage. In order to obtain the FTIR spectra of the AuNRs and PtNCs with the capping ligands of TSC, PVP, DEA or CTAB (or CTAC), the nanocrystals were centrifuged, washed with H_2_O twice to remove any free or physically adsorbed capping ligands, and redispersed in H_2_O (Au, ~ 5 mM; Pt, ~ 8 mM). Then, 20 µL of the sol was dropped onto the sample stage, dried at room temperature, and subjected to the FTIR analysis. In order to obtain the FTIR spectrum of the OAm-capped PtNCs, the PtNCs were centrifuged, washed with toluene twice to remove any free or physically adsorbed OAm, and redispersed in toluene (Pt, ~ 8 mM). Then, 20 µL of the sol was dropped onto the sample stage, dried thoroughly at ~ 50 °C, and subjected to the FTIR analysis. In all measurements, the FTIR signals from the environmental CO_2_ (at ~ 2336 and 2380 cm^–1^) were recorded for rough evaluation of the signal intensity (or the signal-to-noise ratio) from the capping ligands of the metal nanocrystals. (5) Nuclear magnetic resonance (NMR) was measured on AVANCE III 400MHz NMR spectrometer. NMR is a powerful and sensitive tool to characterize the surface-bound ligands on the metal nanocrystals. To ensure that the spectra contain only the information of surface-bound ligands, the nanocrystals were washed thoroughly to remove free capping ligands before the analysis. The ^1^H NMR spectra of pure chemicals of CTAB, PVP, DEA and TSC were recorded by dissolving the pure chemicals in D_2_O. To obtain the ^1^H NMR spectra of the AuNRs with the respective capping ligands, the AuNRs were centrifuged at different stages of the ligand exchange, washed with H_2_O for 4 times, and redispersed in D_2_O. By the repetitive washing, the free capping ligands were removed. The AuNRs were concentrated (Au concentration ~ 125 mM, volume ~ 400 µL) to obtain strong ^1^H NMR signals.

**Table S1.** Detailed binding energies of CTA^+^, VP, DEA, DEA-H^+^, and TSC adsorbed on Au (110) surfaces.

| Adsorbates | Binding Energy / eV |
| --- | --- |
| Au–CTA | –0.973 |
| Au–VP | –1.074 |
| Au–DEA | –1.331 |
| Au–DEA-H^+^ | –0.716 |
| Au–TSC | –0.896 |


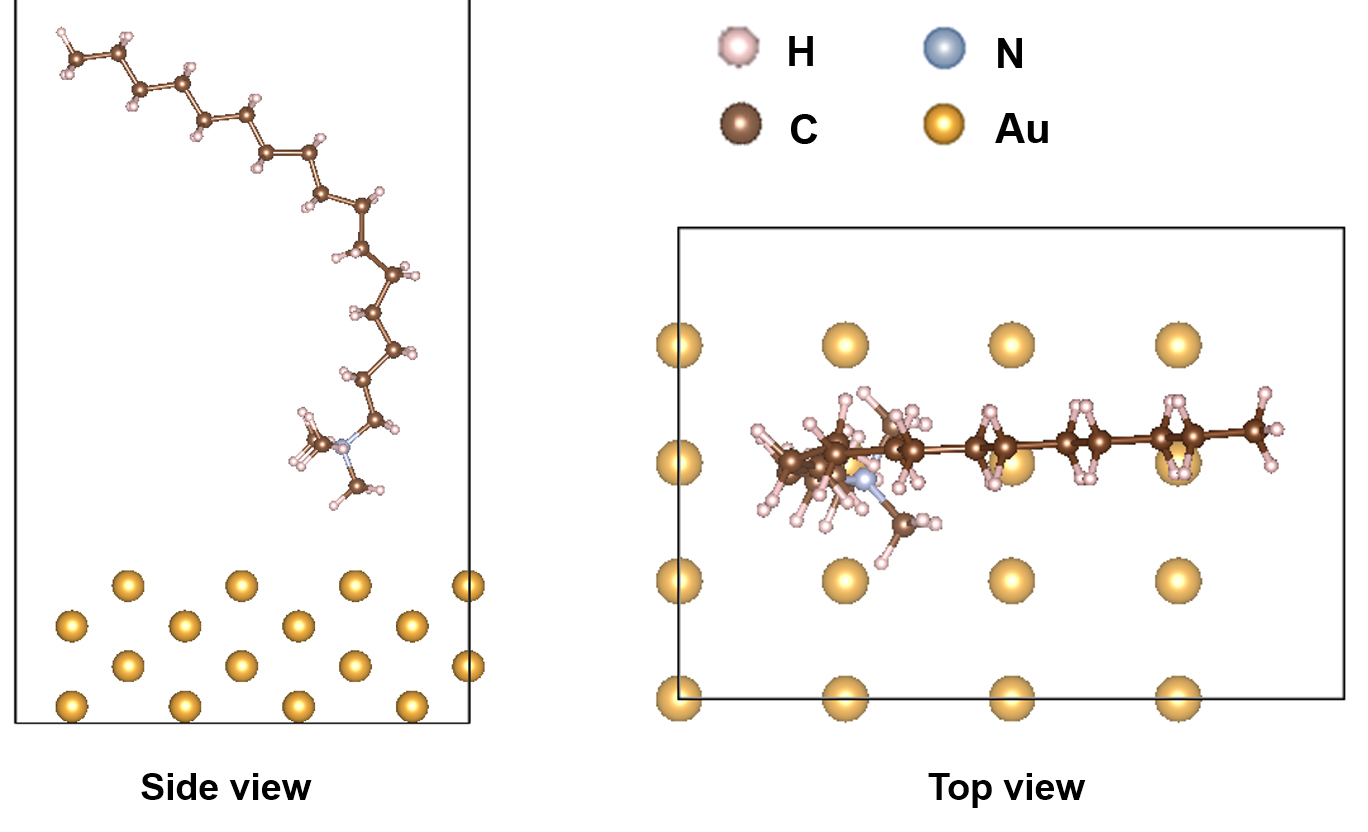


**Figure S1.** Optimized structure of CTA adsorbed on the Au (110) surface.


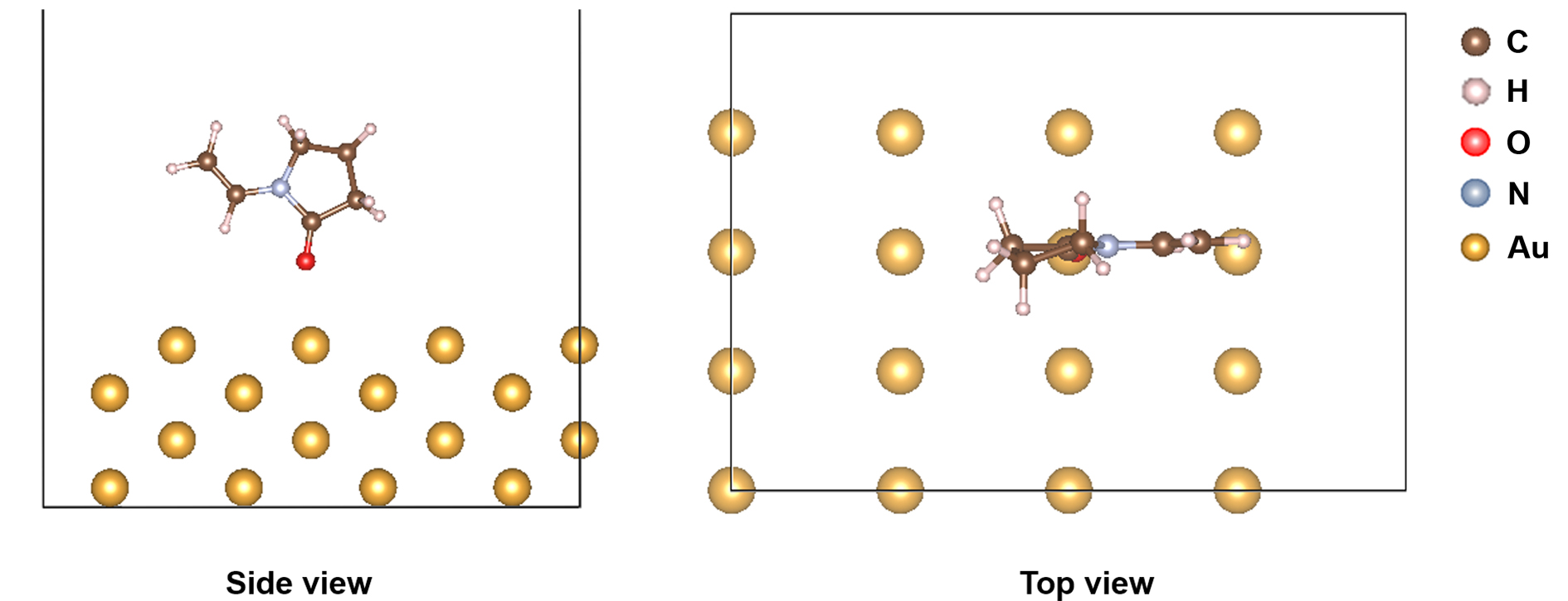


**Figure S2.** Optimized structure of VP (as a simplified model of PVP) adsorbed on the Au (110) surface.


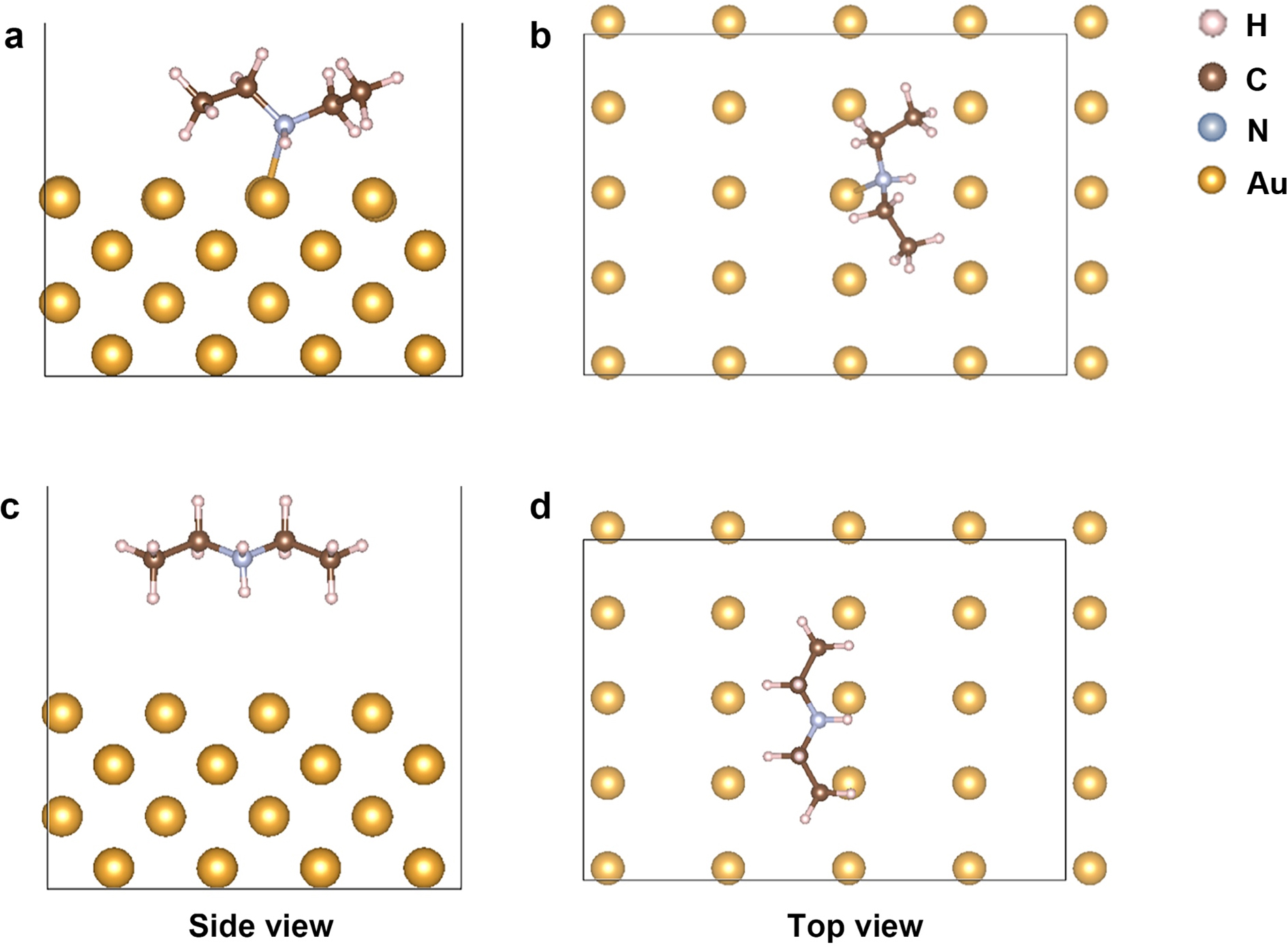


**Figure S3.** Optimized structures of DEA and DEA-H^+^ adsorbed on the Au (110) surface. (a, b) Au–DEA, (c, d) Au–DEA-H^+^, respectively.


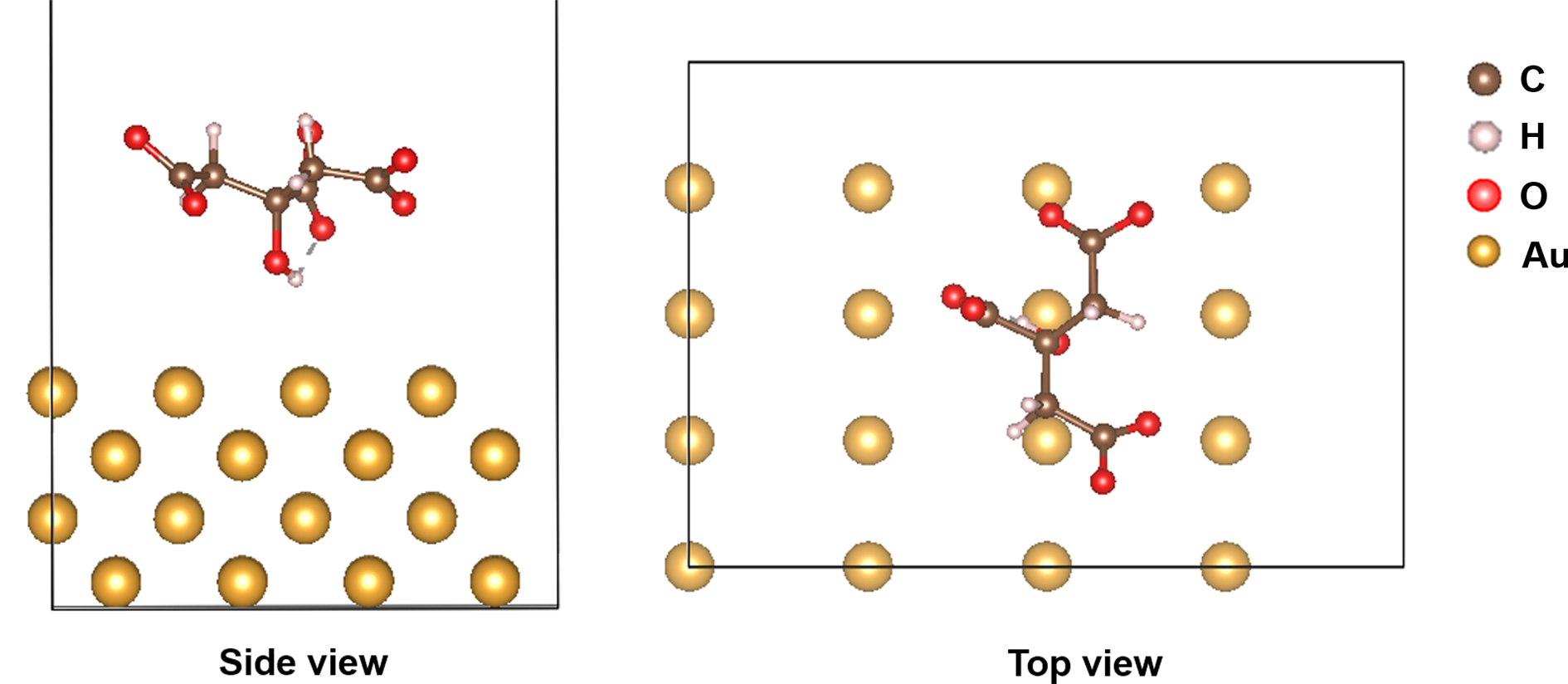

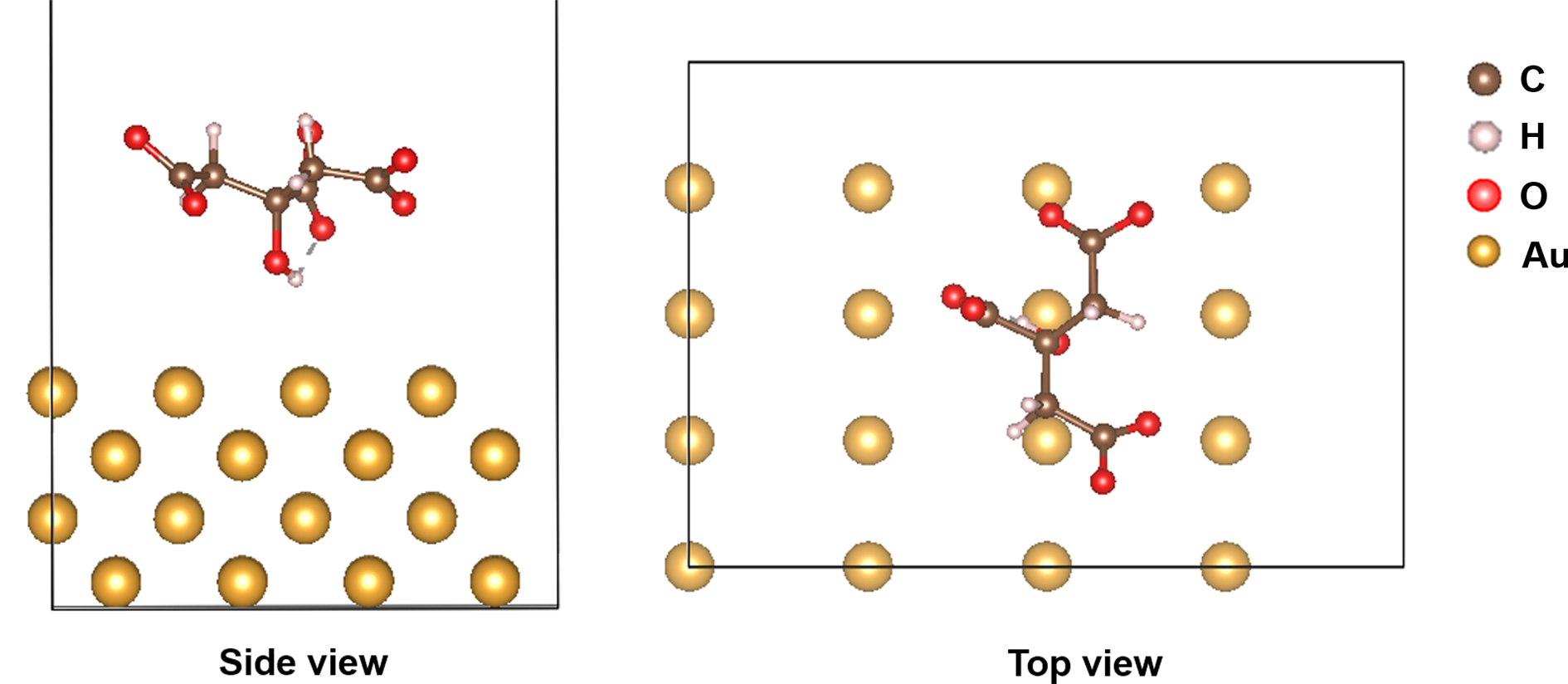


**Figure S4.** Optimized structure of TSC adsorbed on the Au (110) surface.


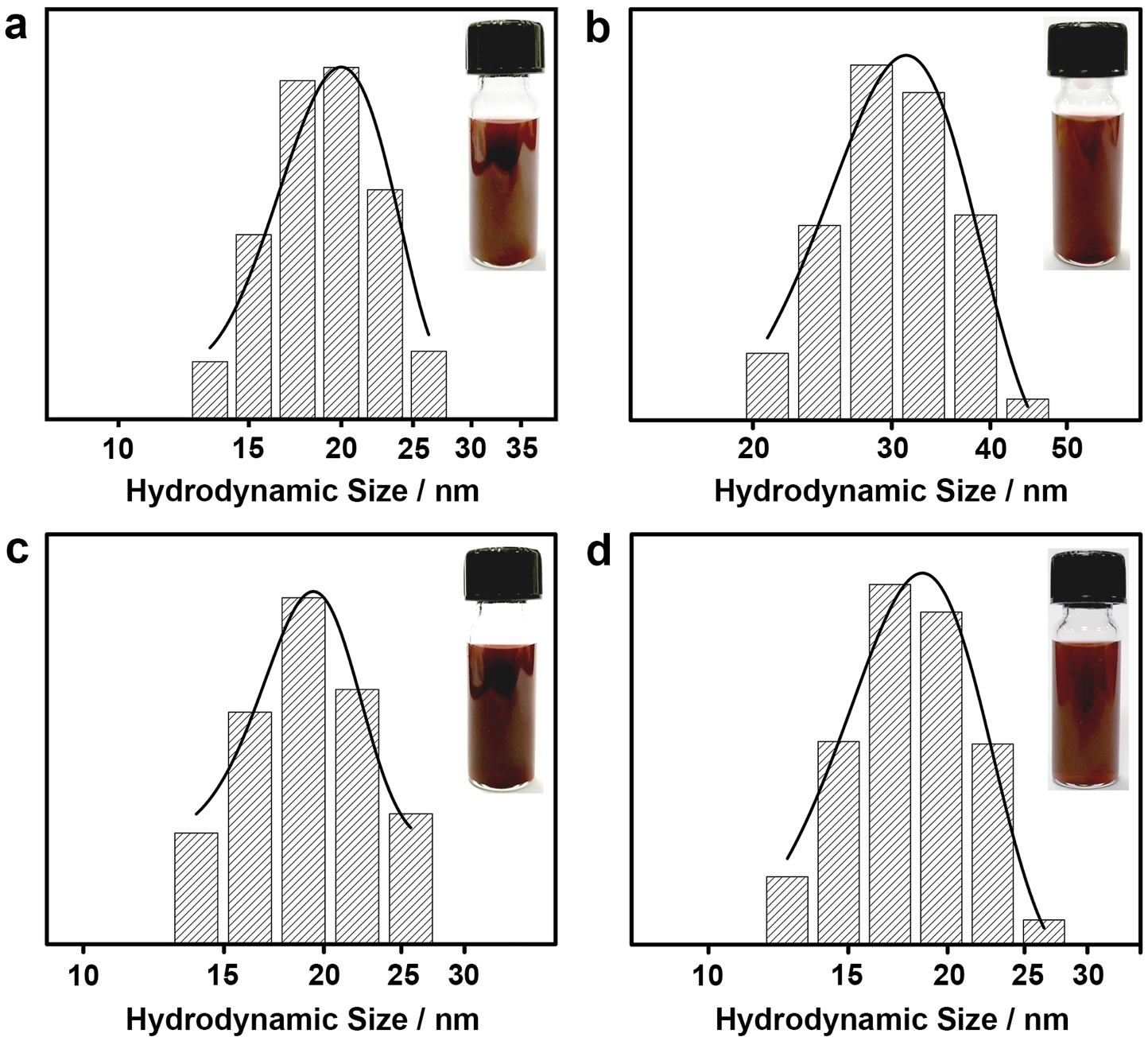


**Figure S5.** Hydrodynamic sizes of the AuNRs obtained at different stages of the ligand exchange, measured by dynamic light scattering (DLS). (a–d) CTAB, PVP, DEA, and TSC-capped AuNRs, respectively. The inset shows the corresponding photographs of the colloids of the AuNRs.

**Discussion:** The AuNRs with the capping ligand of native CTAB showed an initial hydrodynamic size of ~ 19 nm (Figure S5a). After the ligand exchange with PVP, the hydrodynamic size of the AuNRs increased to ~ 30 nm, which could be attributed to the thick hydration shells associated with the polymeric PVP around the AuNRs (Figure S5b). After the further ligand exchange with DEA, the hydrodynamic size of the AuNRs shifted back to ~ 19 nm, confirming the effective detachment of the polymeric PVP from the surface of the AuNRs (Figure S5c). This hydrodynamic size was virtually unchanged when TSC-capped AuNRs were finally obtained (Figure S5d). The DLS results confirmed the retention of the colloidal property of the AuNRs during the whole ligand exchange process.


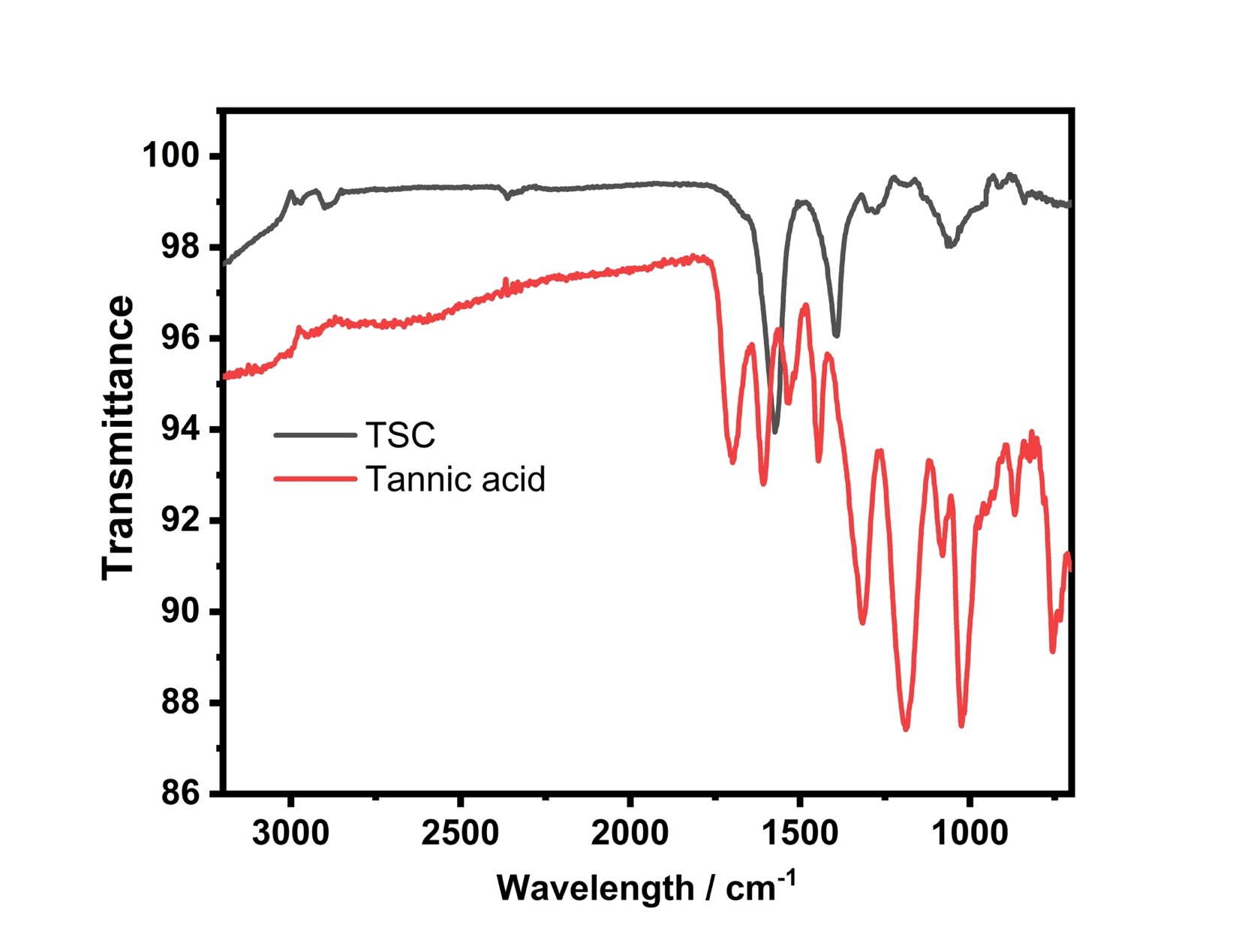


**Figure S6.** FTIR spectra of TSC and tannic acid (TA).


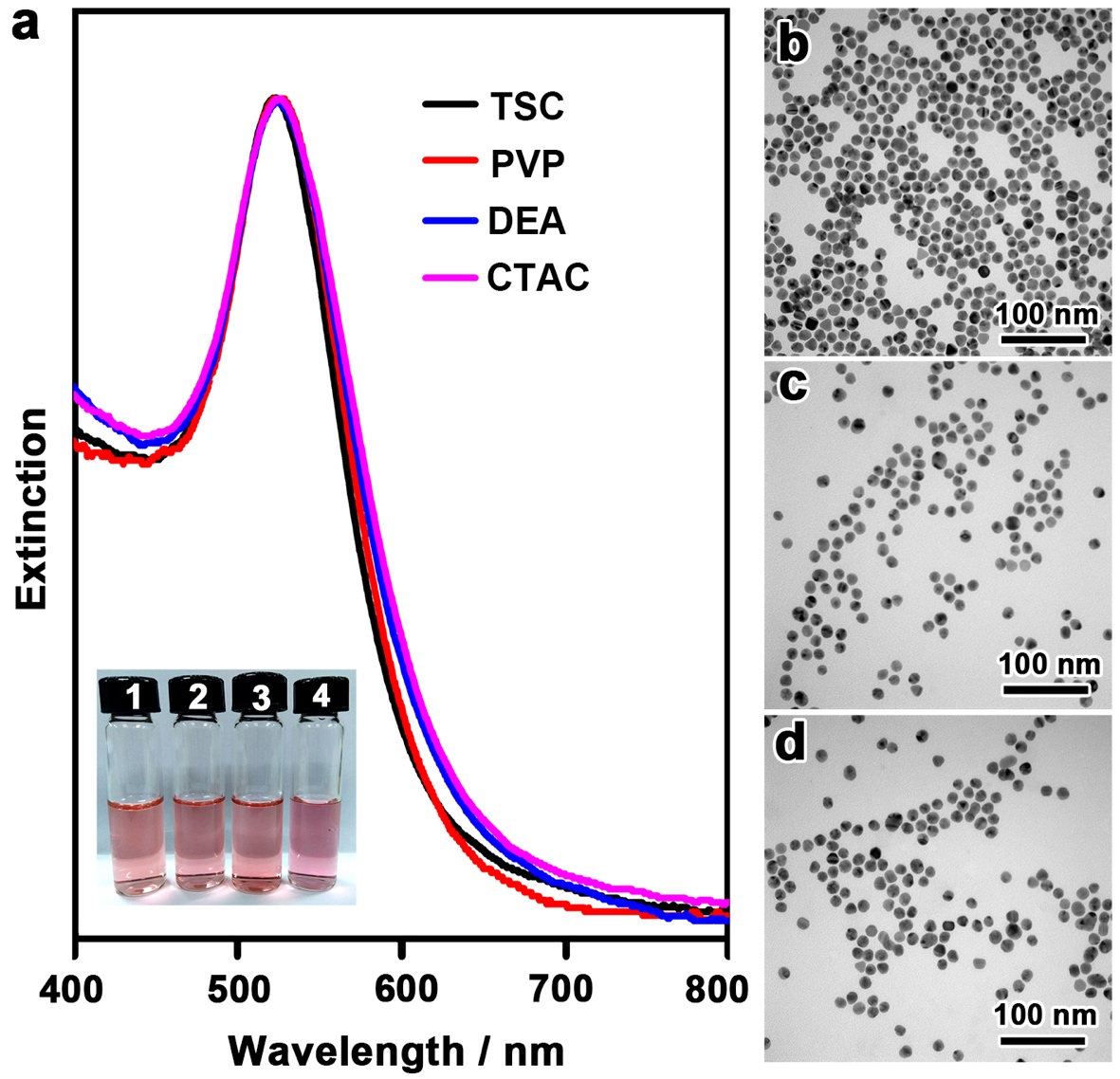


**Figure S7.** Ligand exchange of AuNSs to replace the native TSC by CTAC. (a) UV-vis spectra of the AuNSs capped with different ligands: TSC, PVP, DEA, and CTAC. Inset show photographs of the AuNSs (1–4: AuNSs with the capping ligand of TSC, PVP, DEA, and CTAC, respectively). (b–d) TEM images of the AuNSs with the capping ligand of TSC, PVP, and DEA, respectively (TEM image of the CATC-capped AuNSs, see Figure 4b).


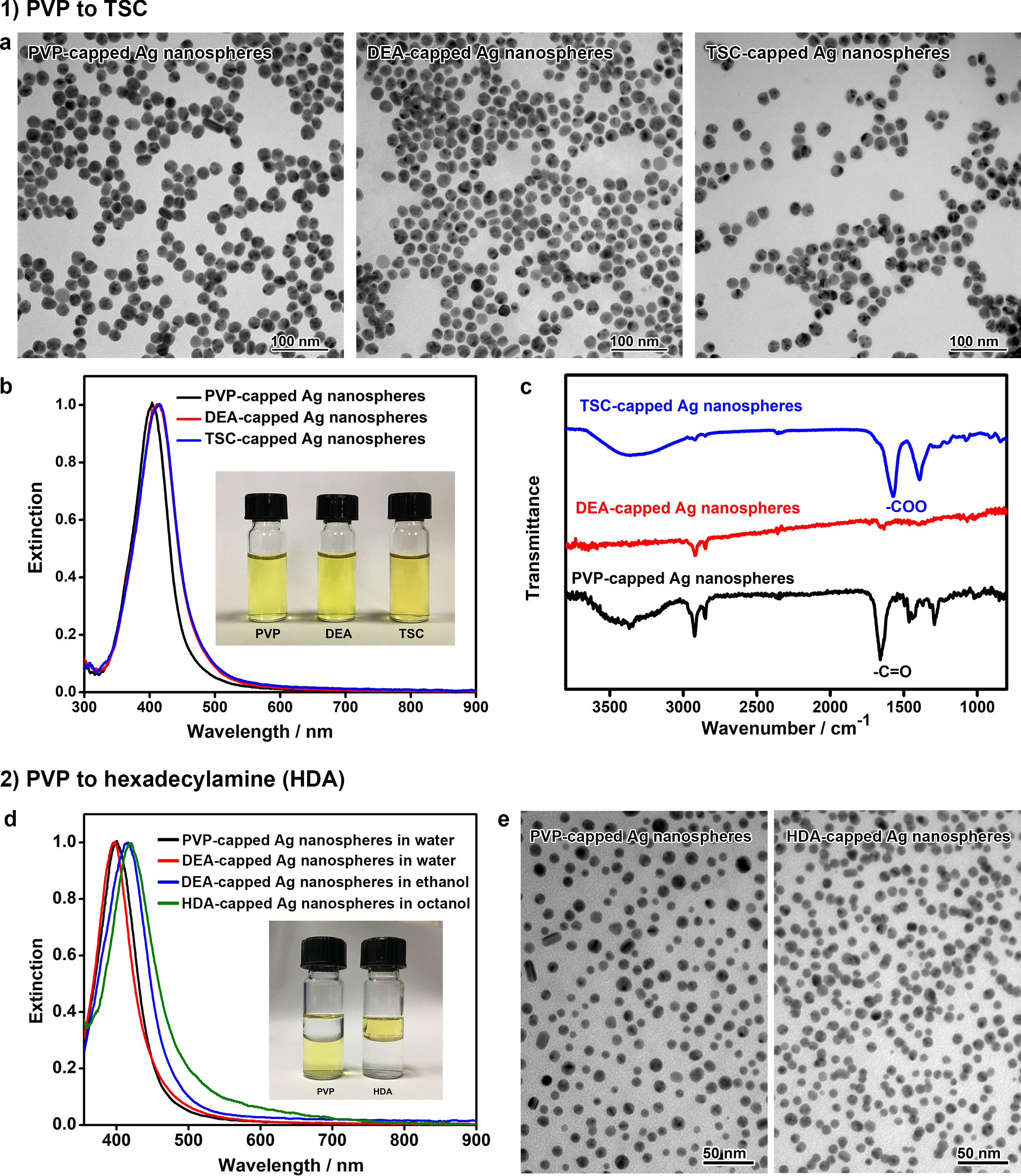


**Figure S8.** Applicability of the ligand-exchange strategy to Ag nanospheres. (a–c) Ligand exchange of Ag nanospheres to replace native PVP by TSC. The PVP-capped Ag nanospheres (~20 nm) were synthesized by a previously reported protocol.^3^ In a typical ligand exchange, 500 μL of DEA and 1 mL of the PVP-capped Ag nanospheres were added to 9 mL of H_2_O (temperature, 60 °C). After 30 min, DEA-capped Ag nanospheres were obtained by centrifugation, washed with H_2_O, and redispersed in 4 mL of H_2_O. To this solution were then added 2 mL of TSC (0.034 M) and 4 mL of tannic acid (1 wt%) (temperature, 60 °C). After 30 min, TSC-capped Ag nanospheres were obtained by centrifugation, washed with H_2_O, and redispersed in H_2_O. (a) TEM images of the Ag nanospheres with different capping ligands. (b) UV-vis spectra of the Ag nanospheres with different capping ligands, confirming the high dispersity of the colloids. Inset: Photographs of the respective colloids. (c) FTIR spectra of Ag nanospheres, confirming complete removal of the prior capping ligands and successful adsorption of new ligands in each step. (d, e) Ligand exchange of Ag nanospheres to replace native PVP by hexadecylamine (HDA). The PVP-capped Ag nanospheres (~ 10 nm) were first synthesized and then the ligand of PVP was exchanged by DEA. The DEA-capped Ag nanospheres were collected by centrifugation and redispersed in 5 mL of ethanol. To this solution were then added 1.6 mL of HDA (0.05 M in ethanol) and 3.2 mL of tannic acid (1 wt%). After 30 min at room temperature, HDA-capped Ag nanospheres were collected by centrifugation and redispersed in octanol. (d) UV-vis spectra of the Ag nanospheres with different capping ligands, confirming the high dispersity of the colloids. The obvious shift of the spectra can be attributed to a change in the capping ligand and the solvent. Inset: Photographs of the respective colloids (top phase, octanol; bottom phase, water). The ligand exchange leads to a transfer of the Ag nanospheres from an aqueous phase to an oil phase. (e) TEM images of the Ag nanospheres with the capping ligands of PVP and HDA.


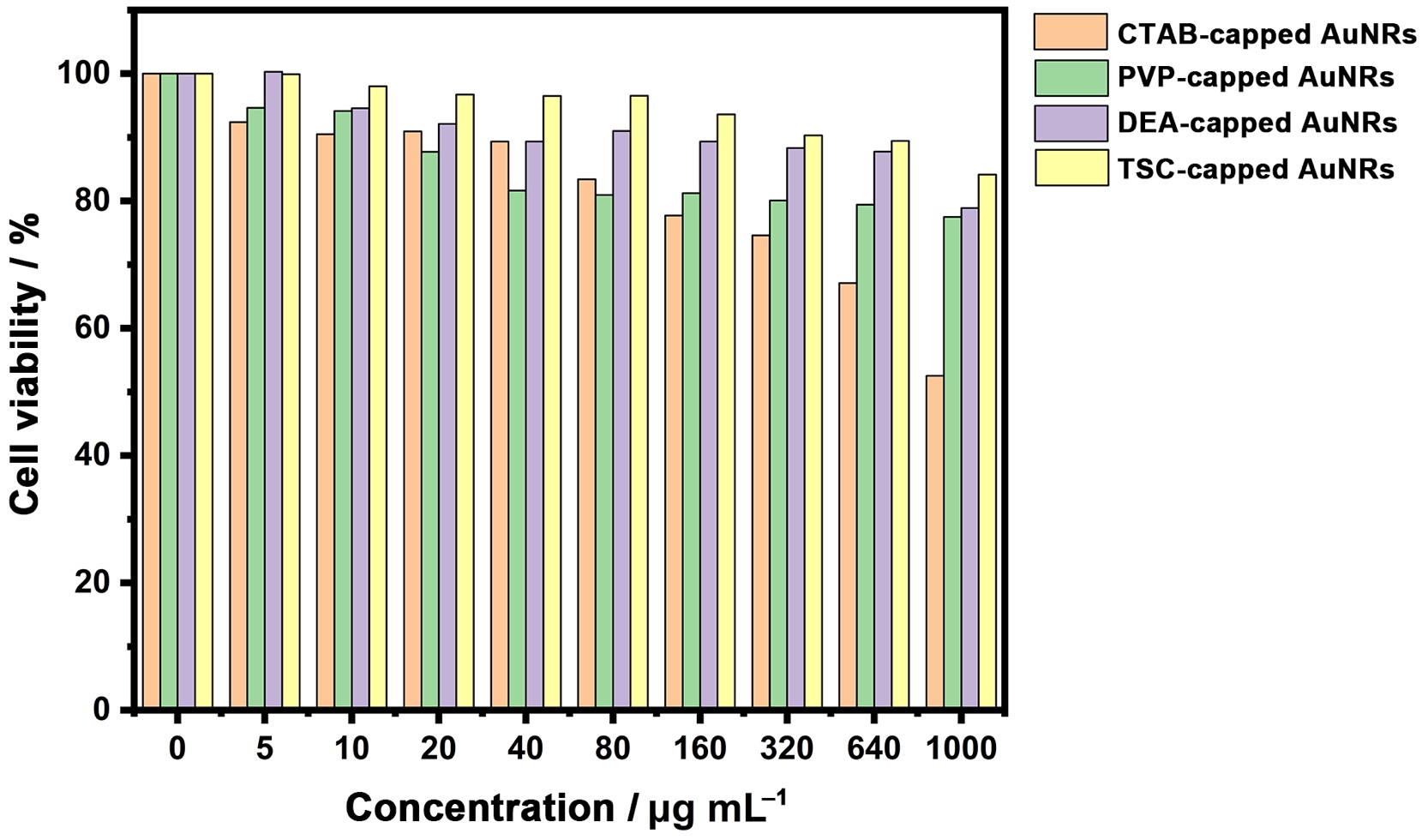


**Figure S9.** Biotoxicity of the AuNRs with different capping ligands and concentrations. This figure shows the viability of the mouse fibroblasts L929 cells after incubation with the AuNRs for 1 day.


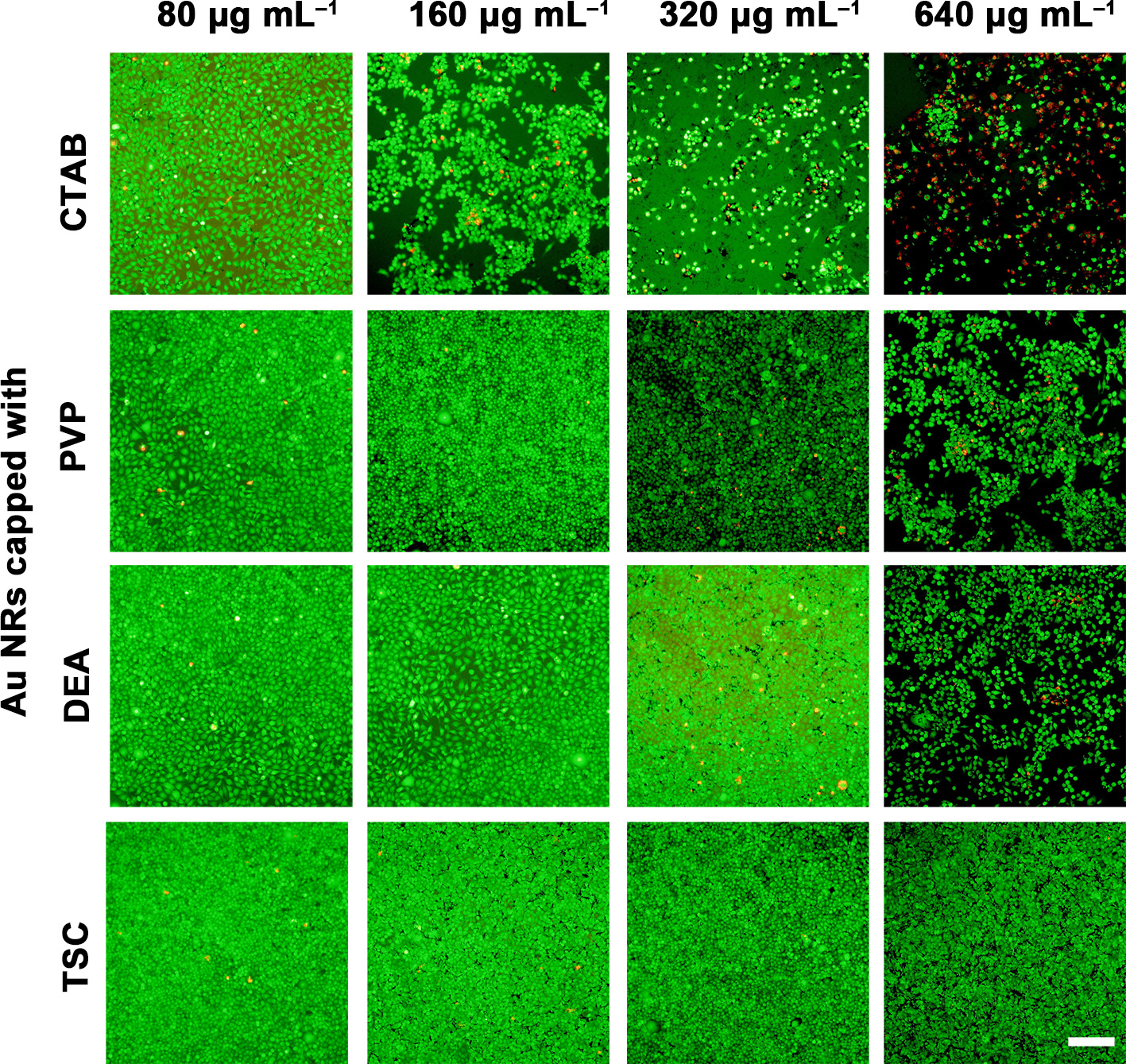


**Figure S10.** Fluorescent images showing the LIVE/DEAD staining of L929 cells after incubation with AuNRs with different capping ligands and concentrations for 3 days (live cells: green; dead cells: red; scale bar, 200 μm).


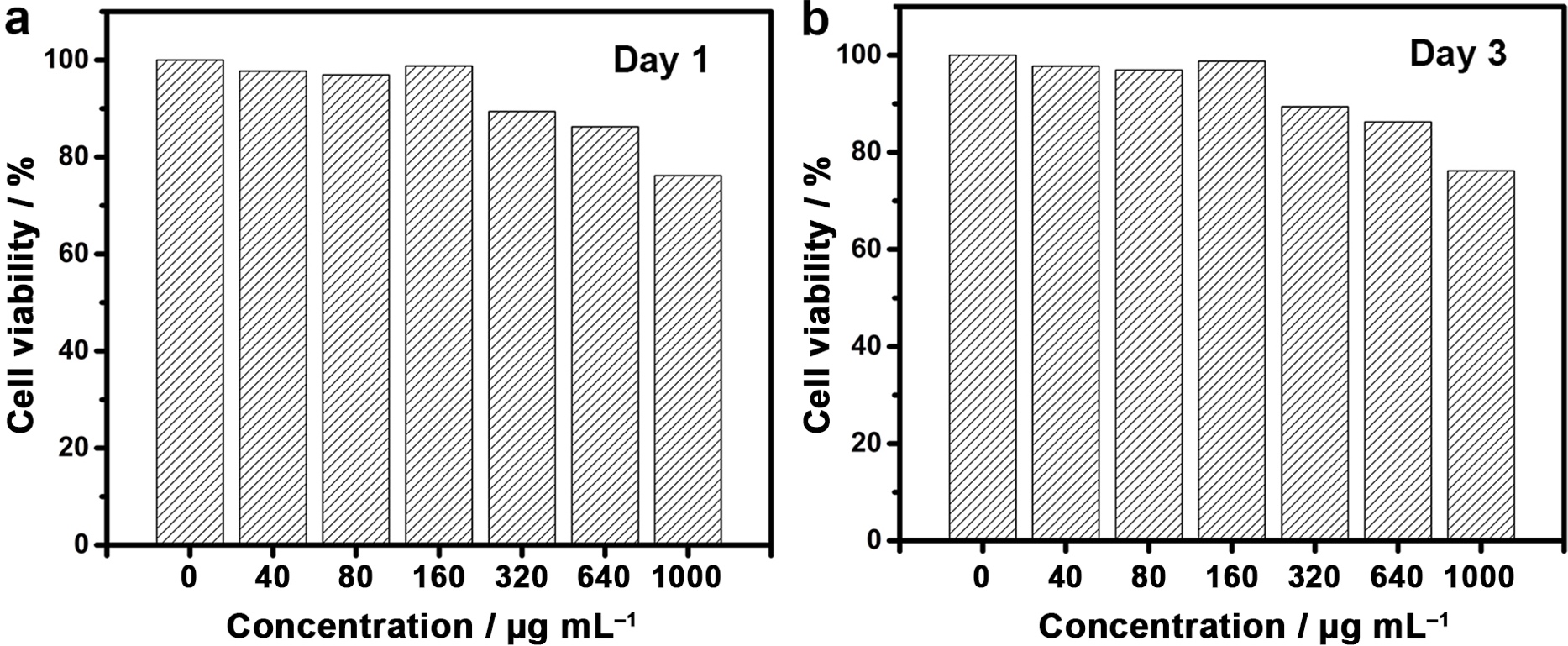


**Figure S11.** Biotoxicity of the TSC-capped AuNRs at different concentrations, showing the viability of the human malignant melanoma cell line A375 after incubation with the AuNRs for 1 day (a) and 3 days (b).


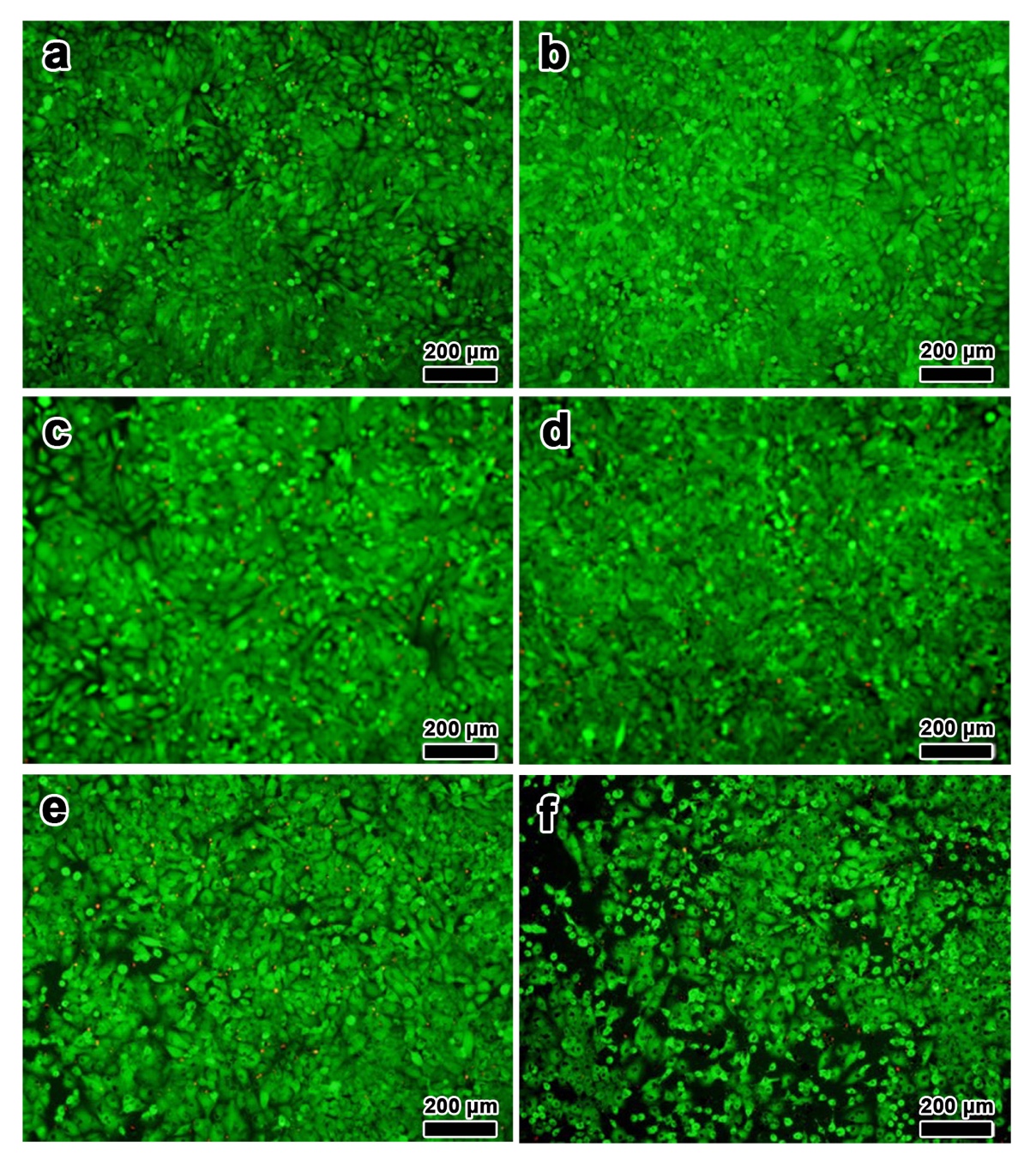


**Figure S12.** Fluorescent images showing the LIVE/DEAD staining of A375 cells after incubation with TSC-capped AuNRs of different concentrations for 3 days: (a) 0 μg mL^–1^; (b) 40 μg mL^–1^; (c) 80 μg mL^–1^; (d) 160 μg mL^–1^; (e) 320 μg mL^–1^; and (f) 640 μg mL^–1^. Live cells: green; dead cells: red.

**References:**

[1] G. Frens, “Controlled nucleation for the regulation of the particle size in monodisperse gold suspensions,” *Nature: Phys. Sci.*, vol. 241, no. 20, pp. 20-22, 1973.

[2] C. Wang; H. Daimon; Y. Lee; J. Kim; S. Sun, “Synthesis of monodisperse Pt nanocubes and their enhanced catalysis for oxygen reduction,” *Journal of the American Chemical Society*, vol. 129, no. 22, pp. 6974-6975, 2007.

[3] X. Liu; Y. Yin; C. Gao, “Size-tailored synthesis of silver quasi-nanospheres by kinetically controlled seeded growth,” *Langmuir*, vol. 29, no. 33, pp. 10559-10565, 2013.
